# Minimum effective volume of ropivacaine for ultrasound-guided supra-inguinal fascia iliaca compartment block

**DOI:** 10.1038/s41598-020-79059-7

**Published:** 2020-12-14

**Authors:** Kumiko Yamada, Shinichi Inomata, Shigeyuki Saito

**Affiliations:** 1 Department of Anesthesiology, Tsukuba Gakuen Hospital, Tsukuba, Japan; 2 Division of Clinical Medicine, Department of Anesthesiology, Faculty of Medicine, University of Tsukuba, University of Tsukuba Hospital, Tsukuba, Japan

**Keywords:** Anatomy, Medical research

## Abstract

Supra inguinal fascia iliaca compartment block (FICB) is increasingly used in elderly patients with hip fractures. However, the minimum effective volume of local anesthetics required for ultrasound-guided supra-inguinal FICB has not been determined. With ethical committee approval and written informed consent from patients, we studied 21 consecutive patients of ASA physical status I–III undergoing surgery for hip fracture who met the inclusion criteria. Blocks were performed before going to the operation room. We determined the injection volumes of 0.25% ropivacaine for consecutive patients from the preceding patient's outcome. The initial volume was 30 ml. The testing interval was set at 10 ml, and the lowest volume was 5 ml. An effective block was defined as loss of sensation of pinprick in the territory of the femoral nerve and lateral cutaneous nerve of the thigh 30 min after the injection. The aim of this study was to determine the 50% effective volume (EV_50_) and the 95% effective volume (EV_95_) of 0.25% ropivacaine for ultrasound-guided supra-inguinal FICB using Logistic regression analysis. EV_50_ and EV_95_ of 0.25% ropivacaine for ultrasound-guided supra-inguinal FICB calculated with logistic regression analysis were 15.01 ml (95% confidence interval, 6.53–22.99 ml) and 26.99 ml (95% confidence interval, 20.54–84.09 ml), respectively. EV_50_ and EV_95_ of 0.25% ropivacaine for ultrasound-guided supra-inguinal FICB were 15.01 ml and 26.99 ml, respectively.

**Clinical trial number**: UMIN000027277 (URL https://www.umin.ac.jp/ctr/index-j.htm).

## Introduction

Hip fracture is a common injury in the elderly. Acute pain management in this group of surgical patients is often difficult; multi-modal analgesia is necessary. In patients with hip fracture, the pain management is often treated with opioids. But which are difficult to adjust and are associated with many side effects especially in elderly patients^1,2^.

Regional anesthesia has been used for pain management for hip fractures for quite a long period of time. Among the procedures, fascia iliaca compartment block (FICB) is believed to be advantageous because of its safety and efficacy^3–5^. FICB is effective and easy to administer, it reduces acute pain and the need for opioids, with few treatment-related adverse effects^6^.

The ultrasound in musculoskeletal medicine and pain management is useful and the ultrasound guided nerve block is popular^7,8^.

Dalens and colleagues^9^ first described FICB in 1989. It was originally intended for pediatric patients. In FICB, local anesthetics are injected into the fascia iliaca compartment, then spread to the femoral and lateral cutaneous nerves of the thigh. A modified supra-inguinal approach of FICB was reported in 2007^6^ and clinical randomized trials reported that the supra-inguinal approach provides better analgesia than infra-inguinal approach in patients presenting for total hip arthroplasty^10,11^.

A volume of 20 ml was sufficient to spread to both the femoral nerve and lateral cutaneous nerve of the thigh in ultrasound-guided supra-inguinal FICB in a cadaver study^12^. However, relatively high amounts (30–40 ml) of local anesthetic solution have been used in the clinical reports of supra-inguinal FICB^6,9,10^. The use of the smallest possible volume of local anesthetics to block the major nerves would improve the safety of nerve blocks. The minimum effective volume of local anesthetics required for ultrasound-guided supra-inguinal FICB has not been determined. We designed this prospective study to identify the 50% effective (EV_50_) and the 95% effective (EV_95_) local anesthetics volume of 0.25% ropivacaine required for ultrasound-guided supra-inguinal FICB.

## Materials and methods

This prospective study was conducted at Tsukuba Gakuen Hospital from September 2016 to May 2017 with approved by the Ethical Committee/ Institutional Review Board of Tsukuba Gakuen Hospital in 2013 (No. 12-04). Informed consent was obtained from all patients. The study was conducted in accordance with the principles of the Declaration of Helsinki. Trial registration was performed via University Hospital Medical Information Network Center Clinical Trials Register (registration number: UMIN000027277, principal investigator: Kumiko Yamada, Date of registration: May 2017). The trial was registered after recruitment began because of an error, but the study protocol was not changed.

This study examined patients categorized as physical status I–III according to the American Society of Anesthesiologist (ASA) and undergoing surgery for hip fracture. Of these patients, we excluded those with diabetes mellitus, dementia, anticoagulant therapy, allergy to amide anesthetic drugs, addiction to alcohol or drugs, body mass index (BMI) > 35, absence or existence of laterality of the sensation in the territory of the femoral nerve and/or lateral cutaneous nerves and peripheral neuropathy.

The aim of this study was to determine the 50% effective volume (EV_50_) and the 95% effective volume (EV_95_) of 0.25% ropivacaine for ultrasound-guided supra-inguinal FICB using Logistic regression analysis. We used Dixon’s up-and-down method to determine the number of cases and the minimum and maximum concentrations required for logistic regression analysis^13^. If prior information is available, the method will be efficient if the testing interval is within the range of 0.5 to 2 SD. Efficiency in the use of participants and precision will also be improved by starting dose close to the eventual EV_50_^14^. We set the initial volume at 30 ml and the interval volume at 10 ml based on previous study^15^. Due to ethical considerations, we set the minimum volume at 5 ml not at 0 ml.

### Nerve block technique

Blocks were performed before going to the operation room. In the ward, noninvasive arterial blood pressure, electrocardiography, and pulse oximetry was applied.

All blocks were performed by two anesthesiologists experienced in ultrasound-guided supra-inguinal FICB.

**Figure 1:**
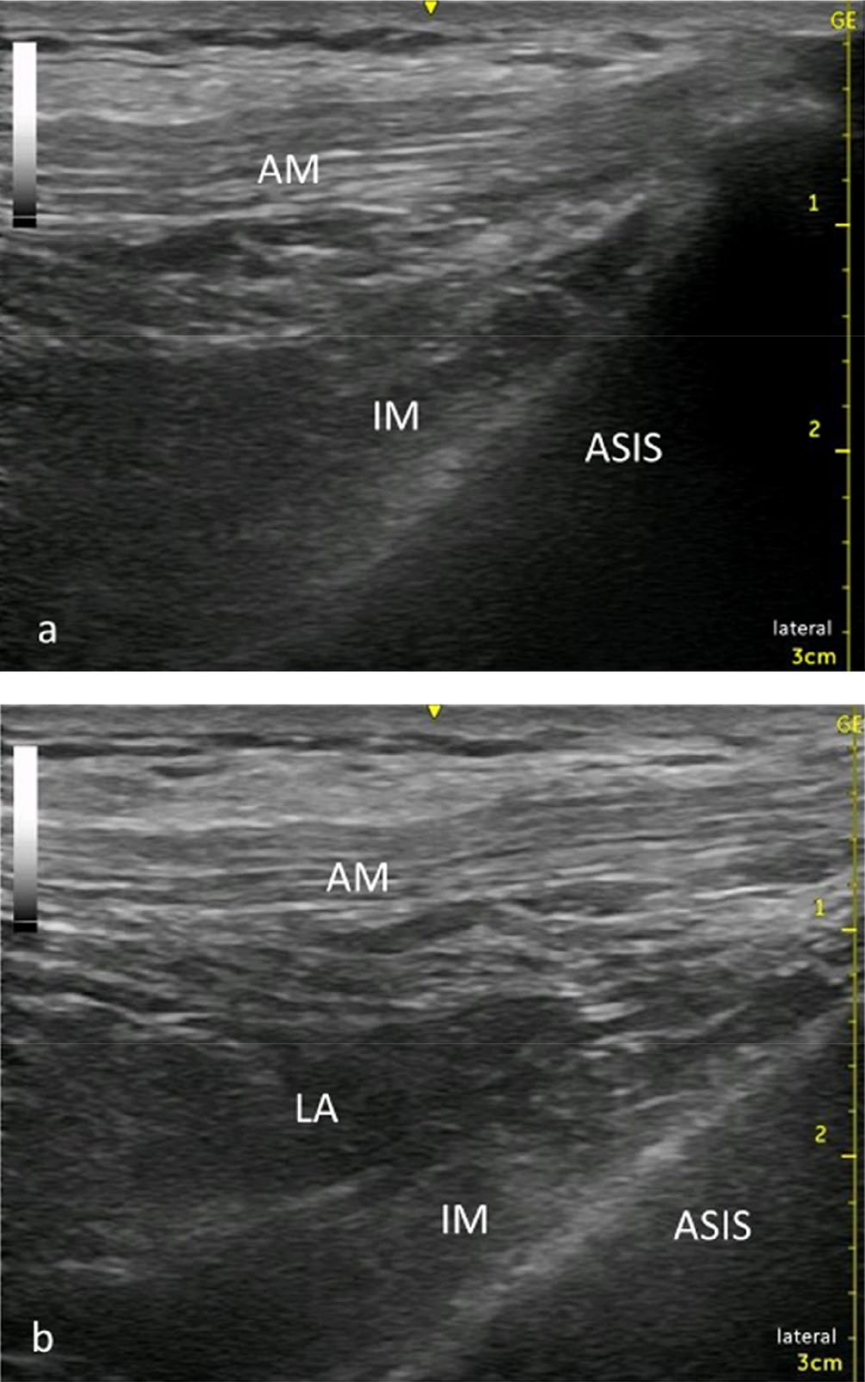
Ultrasound image of pre injection (**a**), ultrasound image of post injection (**b**). *ASIS* Anterior superior iliac spine, *IM* iliacus muscle, *AM* abdominal muscles, *LA* local anesthetic.

The supra-inguinal FICB was performed using a sterile technique with the patient in the supine position. Patients received a FICB under ultrasound guidance using Venue40 (GE Healthcare, Tokyo, Japan) ultrasound unit. After placing a linear probe (5–13 MHz) parallel to the inguinal ligament on the inguinal crease, we found the femoral artery and femoral nerve. From this view, we rotated the probe 90°, making the probe parallel to the vertebral axis. The probe then moved laterally until the anterior superior iliac spine was imaged, and the iliac muscle and abdominal muscles were identified. The medial end of the transducer was rotated to the umbilicus. In this position, a 60-mm needle (23G, Cathelin needle; Terumo Corp, Tokyo, Japan) was introduced at the transducer caudal edge. Using the in-plane approach, the fascia iliaca was penetrated and hydro dissected, separating the fascia iliaca from the iliac muscle. The predetermined volume of 0.25% ropivacaine was injected (Fig. 1).

### Nerve block assessment

Block assessment was performed 30 min after the injection. An examiner blinded to the volume evaluated the presence of a sensory blockade in the territory of the femoral nerve and lateral cutaneous nerve of the thigh. An effective block was defined as loss of sensation of pinprick in the territory of the femoral nerve and lateral cutaneous nerve of the thigh 30 min after the injection. Sensory blockade of the femoral nerve was assessed on the anterior aspect of the distal thigh; that of the lateral cutaneous nerve of the thigh was assessed on the lateral aspect of the thigh.

The anesthesia method during surgery was entrusted to a charge anesthesiologist. For postoperative analgesia, patients received celecoxib 100 ~ 200 mg, diclofenac 25 ~ 50 mg, or acetaminophen 300 ~ 1000 mg as needed.

### Statistical analysis

The anticipated number of “failed-complete” pairs was calculated with power analysis based on the results of Sebel et al.^16^ Six pairs of patients are necessary to provide 80% power (1−β = 0.80) with a 5% two-sided type I error (α = 0.05). For our sample size, we continued data sampling until we had obtained 6 or more mid-points of pairs of volume.

Data are presented as means (ranges) as appropriate. The data were analyzed with logistic regression (SAS System, version 6.12, SAS Institute Inc, Cary, NC, USA) to calculate the volume of 0.25% ropivacaine required to produce a successful femoral nerve block and lateral cutaneous nerve block 30 min after local anesthetic injection in 50% (EV_50_) and 95% (EV_95_) of patients with 95% confidence intervals (CIs).

## Results

**Figure 2:**
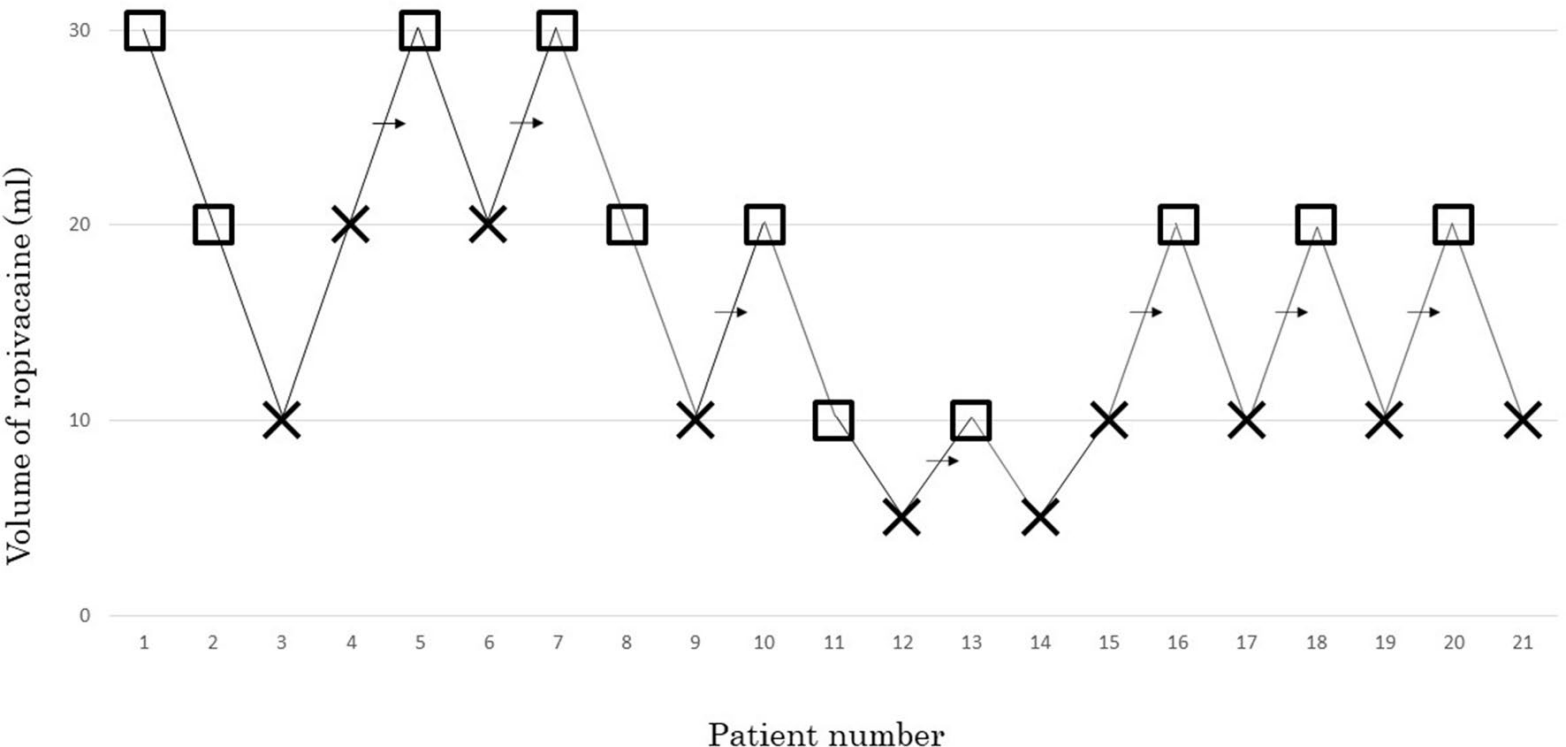
The up-and-down sequence of volumes of ropivacaine 0.25% required for supra-inguinal ultrasound-guided fascia iliaca compartment block to produce effective femoral nerve block and lateral cutaneous nerve block. × failed block, □ = effective block, → mid-point of “failed-effective” pair.

According to Dixon’s up-and-down method, the minimum effective anesthetic volume of 0.25% ropivacaine required for ultrasound-guided supra-inguinal FICB was 15.7 ml. The sequence of positive and negative blocks in the evaluated patients are shown in Fig. 2.

**Figure 3:**
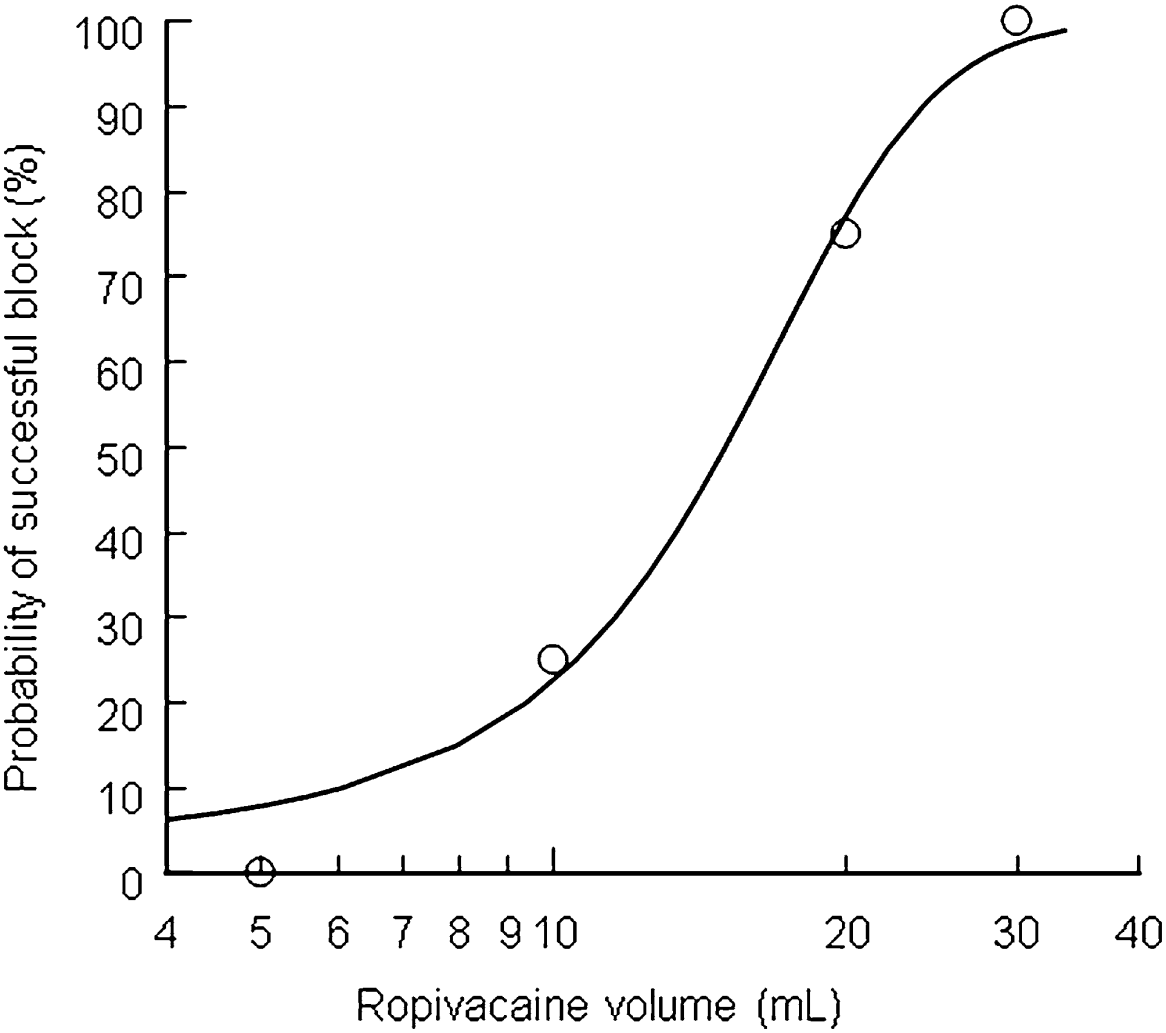
Dose–response curve for ropivacaine plotted from probit analysis. The minimum local analgesic volume of 0.25% ropivacaine for fascia iliaca compartment block was 15.01 ml (95% confidence interval CI − 6.53 ml ~ 22.99 ml), and the 95% effective volume was 26.99 ml (95% confidence interval CI 20.54 ml ~ 84.09.

The EV_50_ and EV_95_ were calculated using logistic regression analysis. The EV_50_ and EV_95_ of 0.25% ropivacaine for ultrasound-guided supra-inguinal FICB to produce effective femoral nerve block and lateral cutaneous nerve block were 15.01 ml (95% confidence interval, 6.53–22.99) and 26.99 ml (95% confidence interval, 20.54–84.09) respectively (Fig. 3).

**Figure 4:**
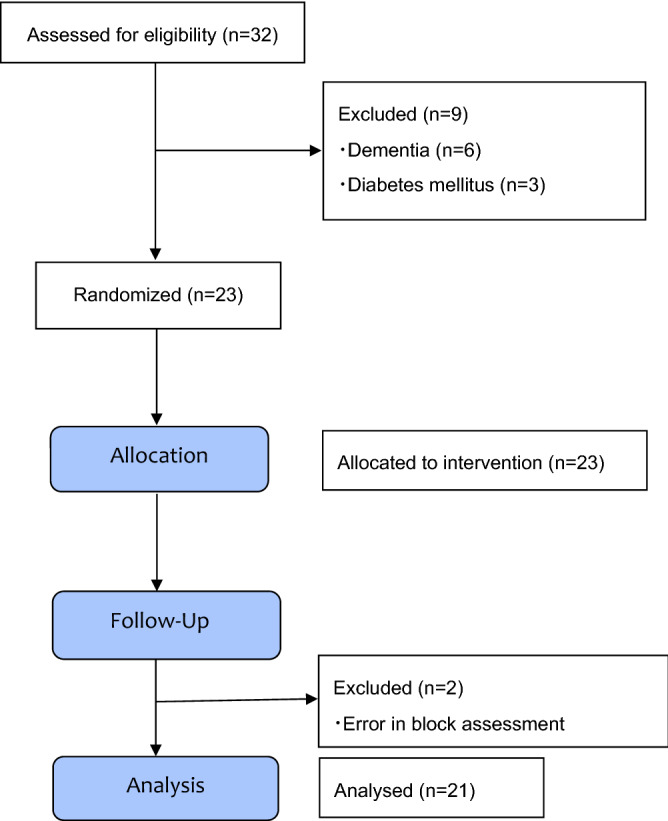
Consolidated standards of reporting trials (CONSORT) chart showing patient recruitment.

In total, 32 patients were enrolled into the study. Figure 4 displays the flow of participants through the study. Nine patients were excluded from the study due to the exclusion criteria described above. To assess the minimum effective anesthetic volume of 0.25% ropivacaine for supra-inguinal FICB, 23 patients who met the inclusion criteria were initially enrolled. We were unable to assess the effect of the block in two patients due to error of the evaluator. A total of 21 patients completed the study and were analyzed. Patient characteristics are shown in Table 1. Table 2 shows the ropivacaine volume and the percentages of patients who had an effective supra-inguinal FICB.

**Table 1:** Patient characteristics.

Patient characteristics	Median (range) or number
Age (year)	83 (43–96)
Sex (m/f)	1/20
Height (cm)	144 (132–160)
Weight (kg)	44 (33–67)
BMI (kg/m^2^)	21.1 (14.2–28.3)

**Table 2:** Percentages of patients who had effective supra-inguinal FICB in each subgroups.

Ropivacaine volume in each subgroup (ml)	Success rate (effective/total)
5	0% (0/2)
10	25% (2/8)
20	75% (6/8)
30	100% (3/3)

*FICB* fascia iliaca compartment block.

No neurological complications were reported at the 24 h follow up.

## Discussion

In this study, the EV_50_ and EV_95_ of 0.25% ropivacaine for ultrasound-guided supra-inguinal FICB to produce effective femoral nerve block and lateral cutaneous nerve block of thigh were 15.01 ml and 26.99 ml, respectively.

A modified supra-inguinal approach for a FICB has been shown to provide superior analgesia for postoperative analgesia in total hip arthroplasty compared to the infra-inguinal approach^6,10,11^, and ultrasound-guided FICB has significantly better success rate compared to the landmark method^17^. Slightly different approaches of supra-inguinal FICB has been reported^12,18,19^. The semi transverse position of the transducer is simple and easy, we used this approach in our study.

This modified supra-inguinal approach creates a more cephalic spread of the local anesthetic agent and more effective blockade of the lumbar plexus than the conventional infra-inguinal approach^12^ and there is a possibility that an effective block can be obtained in a smaller amount. However, there are no dose or volume finding studies relating to supra-inguinal FICB. Most reported dosing regimens in the literature favor a volume of 30–40 ml^6,10,11^. The use of high doses of local anesthetic increases the risk of local anesthetic toxicity. Given that hip fracture patients tend to be underweight, volume for FICB is a critical factor.

Kris Vermeylen et al^20^ reported volume needed to reach femoral nerve, lateral femoral cutaneous nerve, and obturator nerve in cadaver study. Adult cadavers weight between 55 and 72 kg were used in this study. The suggested volume to reach all three nerves was 40 ml, but a volume of 20–30 ml was enough to reach femoral nerve and lateral cutaneous nerve. The result was consistent with our result.

In a previous study, the EV_50_ and EV_95_ for infra-inguinal FICB were found to be 28.8 ml and 34.3 ml, respectively^15^. In that study, the landmark method was used. The use of the supra-inguinal approach is not the only reason for the reduction of solution volume in our results. The combination of the supra-inguinal approach and ultrasound-guided technique probably contributed to the decrease in the required amount of local anesthetic solution.

We did not assessment obturator nerve block in this study. Some authors have discussed that an obturator nerve block dose not alleviate postoperative pain after total hip arthroplasty^21^.

Analgesia of the obturator nerve region may not be essential in hip surgery. Studies of the influence of an obturator nerve blockade on analgesic quality for hip fracture patients are needed in the future. On the other hand, the assessment of obturator nerve block is very difficult because the cutaneous distribution is inconsistent. The skin distribution of the obturator nerve has a big individual difference, and it may incompletely or completely lack. In 57% of patients, there is no contribution to the skin of the obturator nerve^22^. The only method to evaluate obturator nerve was an evaluation of the strength of the hip joint adductor. As for the hip joint adduction, patients with hip joint bone fracture were not able to try this method to cause an intense pain to evaluate obturator nerve block. As a result, we were not able to identify the quantity of the local anesthetic necessary to acquire obturator nerve blocking.

This study has limitations. First, we did not investigate pain scores. Pain scores were collected at predetermined times, but opioids, NSAIDS and acetaminophen were given by the judgment of the medical attendant, making pain scores less reflective of the success of the FICB. Second, we did not investigate the onset time of analgesia and the duration of the analgesia. Levente et al.^23^ demonstrated that the median duration of the analgesia was 48 h with 40 ml of 0.5% ropivacaine. Their result demonstrated prolonged analgesia using a high volume and high concentration technique. A future study on the relationship of the volume of anesthetic solution to the duration and efficacy of analgesia would be helpful. Third, patient physique of this study was small. The mean weight and height of this study population was 44 kg and 144 cm. This maybe does not represent the average weight and height of other population (US, Europe). Considering the difference in physique, we might have used local anesthetic dose per body weight. Forth, there was a bias in the gender ratio in this study (1 male vs 20 females). The number of female hip fracture patients was about 3.7 times larger than the number of male hip fracture patients in Japan, indicating a disparity in gender^24^. The gender difference seems to have increased due to the small number of cases. From the above, study on other population (high BMI, male population) is necessary.

In conclusion, this study demonstrated that the EV_50_ and EV_95_ of 0.25% ropivacaine for ultrasound-guided supra inguinal FICB were 15.01 ml and 26.99 ml, respectively.
